# Medical school department chair performance improvement: A qualitative study

**DOI:** 10.1371/journal.pone.0294927

**Published:** 2024-03-25

**Authors:** Mohammad Mahboubi, Soleiman Ahmady, Azim Mirzazadeh, Afagh Zarei, Hadi Hamidi, Noushin Kohan

**Affiliations:** 1 Department of Medical Education, Smart University of Medical Sciences, Tehran, Iran; 2 Abadan University of Medical Sciences, Abadan, Iran; 3 School of Management and Medical Education, Shahid Beheshti University of Medical Sciences, Tehran, Iran; 4 Faculty of Medicine, Department of Internal Medicine, Tehran University of Medical Sciences, Tehran, Iran; 5 Faculty of Medicine, Department of Medical Education, Birjand University of Medical Sciences, Birjand, Iran; 6 Department of English Language, School of Health Management and Information Sciences, Iran University of Medical Sciences, Tehran, Iran; Maragheh University of Medical Sciences, ISLAMIC REPUBLIC OF IRAN

## Abstract

**Introduction:**

In medical education, department chairs should play a significant role. The present qualitative study was conducted to identify factors that influence the performance improvement of department chairs at medical schools in Iran.

**Methods:**

The study was conducted in Iran in 2022 and used a thematic analysis method. Using a purposeful sampling method, 20 participants were invited to participate, including medical school deans and department chairs. Focus group discussion (FGD) was used for qualitative data gathering. Braun and Clarke’s thematic analysis was used to analyze data.

**Results:**

There were 18 males and 2 females among the participants. The mean age of the participants was 45±4 years. Five overarching themes were formulated: human resource management, organizational behavior management, performance support system, leadership, and financial resources. Also, nine subthemes emerged, including performance evaluation, job and work design, educational and non-educational support, motivational efforts, organization culture, organizational knowledge management, planning for change, and financing.

**Conclusions:**

In this study, we found factors influencing DC performance improvement. Department chairs’ effective performance may have a positive impact on department operations, processes, or outcomes.

## Introduction

"No one plays a larger part in determining the character of higher educational institutions than the department chairman" [1]. The academic department is the main building block of universities in which most of the university labours are done [2]. It also significantly affects the university’s reputation and research excellence. Finally, it serves as a place for faculty and students to interact and share ideas. Wolverton (2005) suggested that “the department chairship is a series of interruptions and interactions with many people at multiple levels of the institution” [3]. Department chairs (DCs) are the agents of activities that shape department members’ attitudes and behaviors. They must be accountable to the upper managers of the organization on the one hand and faculties, staff, and students on the other hand. They play a significant role in managing universities [4].

The performance of DCs is categorized into three areas: roles and relationships, job responsibilities, and associated skills [5]. Studies have offered some responsibilities for DCs including recommendation of faculty members for appointment, promotion, tenure, control of budgets, class schedules and teaching assignments, affecting student interactions with the institution, and establishing and maintaining departmental culture [6].

Department chairs (DCs) are often at the forefront of decision making and have access to resources to help shape the department’s goals and objectives. They are also able to influence the recruitment, hiring, and retention of faculty, as well as the department’s budget and curriculum. Additionally, they can have a significant impact on the department’s culture and atmosphere by setting expectations and fostering an environment that is conducive to learning [5].

Universities are recognized as dynamic units for overcoming changes such as competition, globalization, technology development, and market orientation [7]. Therefore, it is not far-fetched if the roles of DCs, as the heads of the main building blocks of universities, have dramatically changed over time [8] Especially in medical education as a response to healthcare reform prospects, evolving technologies, and knowledge advances. These create significant uncertainties and corresponding responsibilities. The traditional medical education system struggles to keep up with change. It must adapt to meet the changing needs of the health care landscape [9].

Consequently, DCs are likely to be the most influential position in the university to deal with these challenges and manage situations; however, they are often underestimated, as academics with no experience or understanding are assigned to them [10]. This lack of expertise and understanding can lead to DCs being underutilized. This can negatively impact the university’s ability to respond to challenges and manage situations effectively. As such, universities must ensure that experienced and knowledgeable people are appointed to the DCs role.

In the study by Lieff et al. (2013), DCs’ performance is most influenced by their access to a supportive and comprehensive network [11]. And the transition from a faculty member to a department chair is from a ‘‘collegial, discipline-based world to a hierarchical, university-based reality” [12].

DCs are faced with many challenges that are context-dependent when managing challenges, which requires knowledge, experience, and development [13]. DCs’ performance must be studied in different areas of the world [14]. However, there are few studies that emphasize DCs’ roles or responsibilities. There is little known about factors influencing DC performance improvement in medical schools. As such, more research is needed to better understand the key factors that can influence performance improvement.

Also, it is important to mention that DC’s role, responsibilities, and performance vary depending on the culture. Consequently, academic departments can follow different leadership styles, decision-making procedures, and communication practices according to departmental rules. The impact of cultural differences on DC performance in universities must therefore be taken into account. Using a qualitative approach, we identified factors influencing department chairs’ performance improvement at Iranian medical schools.

## Methods

The study was conducted in Iran in 2022 and used a thematic analysis method. Virtual University of Medical Sciences granted approval for this research through the research ethics committee with the registration number IR.VUMS.REC.1400.023.

Using a purposeful sampling method, 20 participants were invited to participate, including medical school deans and department chairs. Incorporating both chairs and deans into focus groups provided valuable insights into DC effectiveness. There is a difference between Deans and Chairs as far as their responsibilities and work experiences are concerned. In this study, it can be useful to identify potential areas of agreement and disagreement to better understand the phenomenon’s complexity. Focus group discussion (FGD) was used for qualitative data gathering. In this study, two group discussion sessions were held to ask participant opinions. An invitation E-mail containing the purpose of the FGDs was sent to the participants. All participants agreed to attend FGDs at a determined time and location.

Initially, the facilitator discussed the ground rules of FGD in terms of respect and confidentiality.

Informed consent was obtained from all participants. They were informed that they could withdraw from the study at any time without consequences. Moreover, permission was obtained for recording the interviews. Confidentiality was maintained throughout all data collection and analysis steps. The sessions continued until ideas were saturated: no one added anything new. Participants answered some questions based on the interview guide, such as: "What are the factors affecting the department chair’s performance improvement?", "How can academic leaders help department chairs perform better?", and ’’What are strategies for improving department chairs’ performance through policy implementation?".The aim of the questions was to better understand the opinions of the participants, as well as to identify any potential strategies or solutions for improving department chairs’ performance. FGD sessions lasted around 90 minutes and were organized and conducted by professional moderators using the discussion guide. Moreover, focus group sessions were audiotaped and transcribed. Thematic analysis was applied to analyse the data based on Braun and Clarke’s procedure [15]. The transcribed data were reviewed several times, and the most significant features were coded. Then, themes were searched and reviewed to be defined and labelled. This process continued until the overarching themes, themes, and sub-themes were formed. After that, the researchers created the thematic map by identifying themes and subthemes [15]. Member checking was used in the data analysis phase with two peers who were experts in educational management (co-authors) to ensure the credibility and trustworthiness of the findings. Furthermore, immersing in the data by the researchers and confirming the findings by participants were the other ways to guarantee the trustworthiness. Written informed consent was obtained from all participants.

## Results

**Fig 1:**
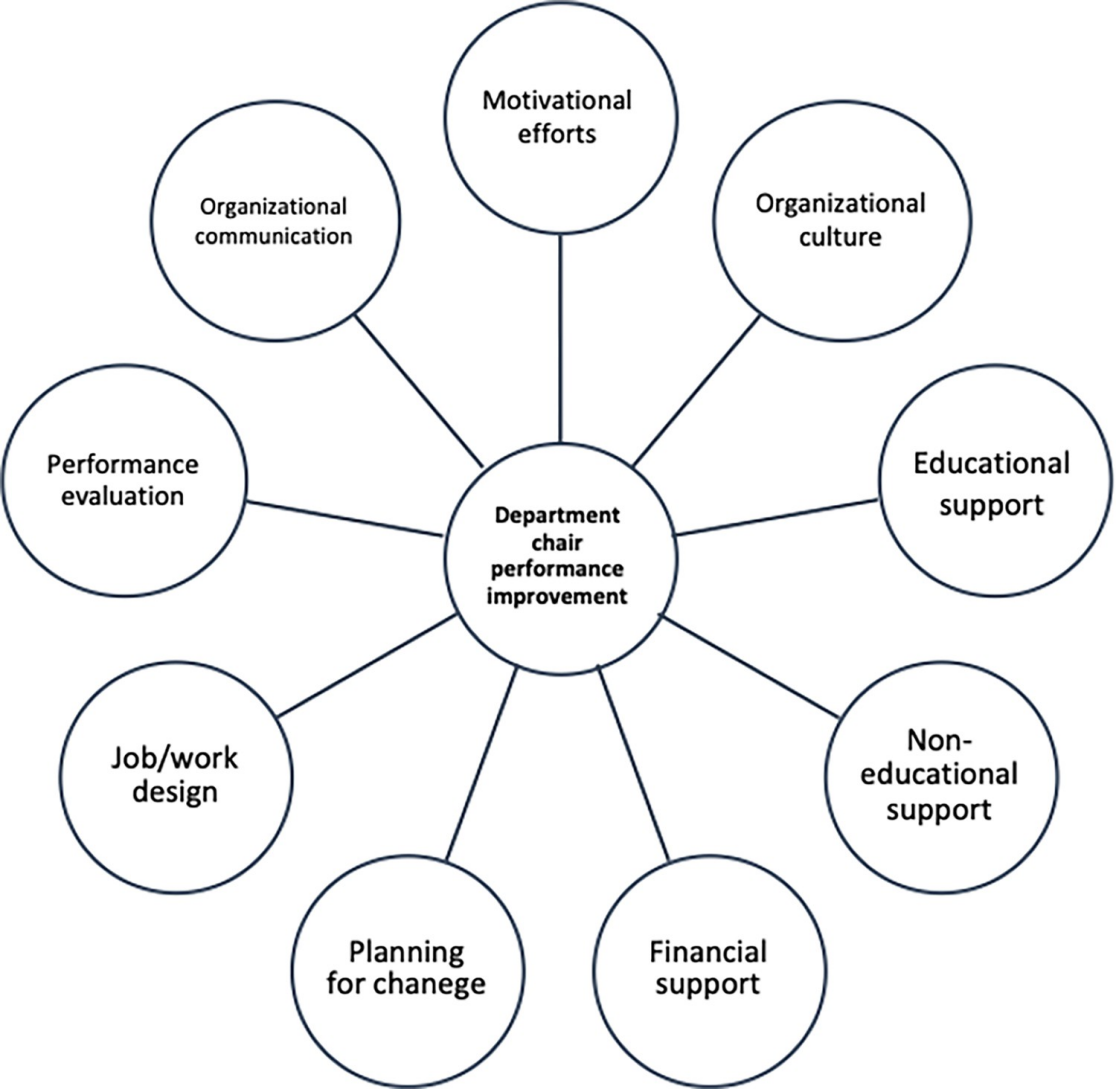
Conceptual framework of the related factors in medical school department chair performance improvement.

There were 18 males and 2 females among the participants. The mean age of the participants was 45±4 years. Based on the thematic analysis, the effective elements on DCs’ performance placed in five overarching themes emerged as follows: 1) Job preparation and expectations factors, 2) organizational behaviour management factors, 3) performance support system, 4) leadership and 5) financial resources development. These five overarching themes included nine themes as well. The nine subthemes were composed of thirty-two sub-themes which can be found in Table 1. The participants’ quotes were also tabulated in front of subthemes they related to in Table 1. The schematic conceptual framework developed accordingly is illustrated in Fig 1.

**Table 1:** Overarching themes and themes, sample of subthemes, and participant quotes.

Overarching themes	Themes	Sample of subthemes	
Job preparation and expectations	Performance evaluation	■ Definition of performance indicators ■ Auditing academic departments	■ *"I think it’s an excellent idea to take the performance evaluation mechanism for DCs seriously*. *It should be considered that the DC has achieved the goals after the program*. *Hence*, *indicators should also be designed principledly*.*"* ■ *"Auditing educational departments is also critical*. *Educational auditing success depends on the extent to which its results are used to develop departments and empower faculty*.*"*
	Job and work design	• Effective selection of academic department chairs • Clarifying the roles and working procedures of faculty members • Establishment of academic department chair task and responsibility • Balance between responsibility and authority • Designing a succession plan	■ *“We first need to identify young people and train them; we must invest in those with leadership and administrative work*.*”* ■ *“In most cases*, *their responsibility doesn’t match their authority*. *If DCs are given authority*, *they can be expected to handle issues*.*"* ■ *“I think managerial work should be separated from executive work*. *That is*, *if we expect DCs’ scientific leadership*, *we should not expect them to be involved in trivial executive responsibilities at the university*.*”* ■ *“We did not clarify our expectations for the faculty members*. *They seem to benefit greatly from a clear understanding of their responsibilities and our expectations*. *In fact*, *if managers want to be transformative and forward-looking*, *they must have a specific job description of their members and ask their ideas about it*.*”* ■ *“It is admirable that there is a selection process*, *but it is obvious that we have to correct the existing process*.*"*
Organizational behaviour management	Organizational culture	■ *Supportive culture* ■ *Justice* ■ *Accountability* ■ *Critique and trust* ■ *Organizational commitment* ■ *Being customer-oriented* ■ *Gratitude* ■ *Teamwork*	■ *"I think there is a distance between faculty and DCs*. *Faculty and DCs are teammates*. *DCs need to support faculty members*, *and DCs need to be supported by the dean and university officials as well*.*”* ■ *"If there is a system that identifies these educational*, *research*, *and scientific resources for colleges and designs an appropriate framework for educational justice*, *it helps strengthen the moral culture in the department to fulfil their role better*.*"* ■ *"Creating an accountability culture is very critical; this means that DCs are committed to the department and the college*, *and thus they consider the organization’s income as their own income*.*”* ■ *"One of the ways to cope with these challenges is to have a culture of critical reflection and trust at the lower and upper levels of the university*.*"* ■ *"If faculty members devote all their time and energy to the university*, *department problems will be solved*.*"* ■ *"It would be very helpful if we showcased educational products in the department and believed in academic marketing*. *We must improve the consumer-oriented culture in all departments*.*"* ■ *"DCs must be appreciated when they do something for the university*. *In my case*, *for example*, *I dealt with many challenges*, *but nobody praised us*.*"* ■ *"I think the teamwork culture in departments is not well-defined*. *We need to strengthen it*.*"*
	Organizational communication	■ Networking of stakeholders ■ Organizational reporting ■ Communication with senior officials ■ Communication with people outside the department	■ *"When it is said that the department is a small college*, *it means that the DC must be in touch with financial managers*, *educational and internationalization deputies*, *and student affairs officials*.*"* ■ *“Reporting is quite significant*. *Senior officials should ask DCs to report on their performances*.*"* ■ *This DC does not have a clear relationship with the university’s educational deputy*. *I think the top manager doesn’t communicate with DCs*, *but they have to*.*"* ■ *"If the department chairs are directly linked to the dean*, *hold common meetings*, *form councils*, *and establish a communication system*, *by taking advantage of these factors*, *they can take their roles more seriously and act effectively*.*"* ■ *“DCs should regard themselves as part of a larger group at the university*. *We can see that their performance will improve*. *They should not think they are separate from the departments*. ■
	Motivational efforts	■ Perception of support ■ Empathic atmosphere ■ Providing funds to departments ■ Informing departments about grants and policies ■ Participation in decision-making	■ *Senior managers must support them*. *It is not enough*. *From my point of view*, *DCs must feel supported by an excellent leader*.*"* ■ *"We must create an atmosphere of empathy in the departments*. *Consequently*, *professionalism*, *friendliness*, *consumer empathy*, *security*, *fairness*, *and efficiency will be essential for the survival of the departments*.*"* ■ *"Providing resources*, *funding for each department*, *and allocation of resources based on staff numbers are critical*.*"* ■ *“We must know grant opportunities*, *grant funds awarded*, *and latest grant policies*.*"* ■ *It would be great to play a crucial role in the university’s strategic thinking process*. *This is a challenge to face*.*"*
Performance support system	Educational support Non-educational support	■ Empowerment of leaders ■ Holding orientation programs ■ Implementing mentoring programs ■ Providing resources and facilities	■ *"Many DCs who participated in the leadership workshops confessed that if they had known about the scientific concepts they learned in these workshops*, *they would have solved many problems and faced challenges better*.*"* ■ *"Holding orientation programs for DCs is necessary*, *and people should be empowered from the beginning*.*"* ■ *"I think the other issue is the need to share experiences*. *It helps us so much*. *We have effective experience and performance in different educational areas that can be transferred to others*.*"* ■ *"Mentoring should be taken seriously in educational departments*, *formally or informally*.*"* ■ *"As DCs*, *we are still engaged in physical space and other problems*. *These problems should be addressed in advance*.*"*
Leadership	Planning for change	■ Needs assessment ■ Defining missions and goals ■ Alignment in planning	■ *“We have to define the mission and those expectations*, *and we have to move toward the mission and those expectations*.*”* ■ *“In order to get the desired results*, *we have to move toward the university mission*. *Needs assessment must be done*.*”* ■ *“If we develop the program*, *it should be in line with the university’s four-year plan*.*"*
Financial resources development	Financing supply	■ Expendable activities	■ *“One problem with executing programs is the resources that guarantee performance*. *There must be both legal and financial mechanisms for the program to be executed*.*”*

### Overarching theme 1: Job preparation and expectations factors

This overarching theme was generated from two themes including job/work design and performance evaluation. The participants mentioned the importance of selecting an appropriate person as well as clarifying the role and responsibilities to improve the performance of DCs. These factors were related to job and work design. In addition, the participants in the FGDs expressed their opinions on how performance evaluation can be improved. They highlighted defining good performance indicators as well as auditing the DCs in the base of these indicators.

### Overarching theme 2: Organizational behaviour management factors

In this regard, three themes were identified. Participants expressed their views about organizational culture, organizational communication factors, as well as the efforts to motivate DCs.

### Overarching theme 3: Performance support system

Educational and non-educational support factors are related to the performance support system*’*

### Overarching theme 4: Leadership

According to the participants, department chairs (DCs) should possess the capacity to lead and be adaptable to change. Participants said DCs need to learn to change and improve their leadership ability.

### Overarching theme 5: Financial resources development

Financial factors were mentioned as a guarantee factor for DC performance in the FGD. From their perspective, DCs need financial resources to execute programs.

The framework shows that higher managers could help DCs through creating appropriate organizational communication, motivational efforts, and creating a positive organizational culture. DCs also need to provide an educational and non-educational support system, leading to performance improvement. Most importantly, they need a university plan to develop their leadership and a financial resource to manage the changes or create a departmental program. Finally, DCs must evaluate and audit using the anticipated indicators defined in advance. However, first they must clarify their expectations and descriptions.

## Discussion

The present study aimed to investigate factors affecting department chairs’ performance improvement. The study findings outweigh those of previous studies since we identified the dimensions of performance improvement from experts’ perspectives in an academic center, focused on both individual and organizational variables, and finally provided a framework that can be easily used in practice. Based on our results, performance evaluation is one of the most critical components of any performance improvement structure. Our findings are consistent with those of other studies reporting that DCs strongly agreed to have a managerial role and responsibility. The performance appraisal system is a prerequisite for ensuring performance success [16,17]. These qualitative findings confirm London (2011)’s suggestion for a performance evaluation system for assessing department chairs’ effectiveness [18]. Furthermore, this study indicated that both organizational job and work design affect academic DC performance. Our findings are consistent with studies that report that well-designed jobs and work have a constructive impact on employees’ motivation and performance; consequently, this improves individual and group organizational performance, such as participation, effective role modelling, and innovative achievement [19,20].

Based on our results, both organizational culture and organizational value could improve DCs’ performance. Organizational culture is "a system of shared meanings held by members who distinguish the organization from other organizations" [21]. These results support the hypothesis that organizational culture is linked to performance. However, management experts claim that successful organizations are those which promote cultural values in line with their strategies [22–24]. In addition, Koesmono (2014) revealed that organizational culture could affect organizational loyalty [25]. Given the findings of this study, organizational culture can improve individual and organizational performance. This result is supported by other studies, which indicate a relationship between strong culture and organizational effectiveness [23–26]. Mullakhmetov et al. (2018) discussed in his study that an effective culture is created by the combination of cultural values and norms. Therefore, culture-related factors affect performance [27].

Based on our results, the development of approaches and techniques for organizational communication, as well as organizational knowledge management in departments, is essential for improving the performance of DCs. These findings further support the idea that an organization with knowledge management strategies will use assets more efficiently and, as a result, will achieve better organizational performance [28]. Inter-organizational communication will facilitate continuous communication with senior faculties and university administrators, leading to a productive culture in which all members of the institution have a common commitment to performance improvement [29]. Our study confirms numerous studies reporting that more motivated staff are more productive and committed [30,31]. Koesmono (2014) revealed that extrinsic and intrinsic motivation significantly influences employees’ performance [25]. It will be desirable for higher-education organizations to provide educational support services for their DCs to improve their performance and productivity with activities such as preparing a forum for the exchange of experiences, developing a formal orientation program for new DCs, and establishing a mentoring system and leadership development programs [32,33].

The results of this study demonstrated that in addition to educational support, other non-educational support services are required (e.g., logistic support). It is in line with those studies suggesting that supportive organizations make sense of self-importance in their staff, rewarding them, and understanding and meeting their demands [34]. According to Eisenberger et al. (1986), organizational support is "employees’ perception of being valued and cared about by their organization" [35].

Since medical universities undergo rapid changes, DCs, as the leaders of this change, play a vital role in the effectiveness of change efforts [13,36]. The present study illustrated that the leadership delivered by the DCs is an essential factor for success. DCs must supervise the department and possess the power to influence the expected faculties’ interactions and lead a supportive culture for them as a leader [37,38]. Based on our literature review, we were surprised to find relatively few empirical studies about the factors associated with DC performance in peer-reviewed journals, the qualitative method is often employed when there are limited empirical publications available on a particular research construct.

According to Columbus et al, Several factors contributed to the advancement of chairs in academic surgery departments, both internal and external, It may prove crucial to promote career achievement and diversity in surgical management roles in the future to emphasize intrinsic strengths, cultivate supportive environments, and emphasize flexibility. It may also be possible to achieve this through workplace restructuring. As a surgical governance organization, encouraging inherent talents, nurturing conducive environments, and focusing on adaptability may be crucial for success and increasing representation [39].

Navaebrahim and Karimi studied the relationship between the skills of DC (technical, human relations, conceptual) and educational quality. Correlation analysis revealed technical and conceptual skills positively correlated with quality, but human relations skills did not correlate. Thus, technical and conceptual abilities of department heads seem to influence educational performance more so than human relations skills. This study investigated the link between managerial competencies and quality, finding technical and conceptual expertise most impactful for improving quality [40].

The study’s strength lies in its diverse sample of participants, from various universities, medical specialties, and experience levels. As a qualitative research has a context-centered nature, which makes generalizability across contexts difficult. Therefore, we believe that the present study provides significant insights regarding this topic, which can be used to formulate a significant "big-picture" view of the performance improvement of department chairs if complemented with similar research in the future, through meta-analysis.

These findings have important implications for medical schools and academic institutions, as they suggest practical ways to improve the functioning and performance of department chairs, ultimately enhancing the quality of medical education and research. Furthermore, the study’s qualitative approach provides a rich and nuanced understanding of the experiences and perspectives educational expert, which can inform future research and policymaking in this area.

## Conclusion

In this study, we found some factors influencing DC performance improvement in medical schools. Overall, we hypothesized that department chairs’ effective performance may benefit department operations, processes, or outcomes. We propose that department chairs should possess strong leadership skills in order to effectively navigate the professional environment. They should build positive relationships with faculty, and remain committed to the university’s mission and vision.
